# Dietary phosphate supplement does not rescue skeletal phenotype in a mouse model for craniometaphyseal dysplasia

**DOI:** 10.1186/s12952-016-0061-0

**Published:** 2016-10-26

**Authors:** Yaling Liu, Eliane H. Dutra, Ernst J. Reichenberger, I-Ping Chen

**Affiliations:** 1 Department of Oral Health and Diagnostic Sciences, School of Dental Medicine, University of Connecticut Health, Farmington, CT 06030 USA; 2 Department of Craniofacial Sciences, School of Dental Medicine, University of Connecticut Health, Farmington, CT 06030 USA; 3 Department of Reconstructive Sciences, School of Dental Medicine, University of Connecticut Health, Farmington, CT 06030 USA; 4 Department of Oral Health and Diagnostic Sciences, University of Connecticut Health (UConn Health), 263 Farmington Avenue, Farmington, CT 06030-3705 USA

**Keywords:** Craniometaphyseal dysplasia, ANKH, FGF23, Phosphate diet

## Abstract

**Background:**

Mutations in the human progressive ankylosis gene (*ANKH; Mus musculus ortholog Ank*) have been identified as cause for craniometaphyseal dysplasia (CMD), characterized by progressive thickening of craniofacial bones and flared metaphyses of long bones. We previously reported a knock-in (KI) mouse model (*Ank* ^KI/KI^) for CMD and showed transiently lower serum phosphate (Pi) as well as significantly higher mRNA levels of fibroblast growth factor 23 (*Fgf23*) in *Ank* ^KI/KI^ mice. FGF23 is secreted by bone and acts in kidney to promote Pi wasting which leads to lower serum Pi levels. Here, we examined whether increasing the Pi level can partially rescue the CMD-like skeletal phenotype by feeding *Ank* ^+/+^ and *Ank* ^KI/KI^ mice with high Pi (1.7 %) diet from birth for 6 weeks. We studied the Pi metabolism in *Ank* ^KI/KI^ mice and CMD patients by examining the Pi regulators FGF23 and parathyroid hormone (PTH).

**Results:**

High Pi diet did not correct CMD-like features, including massive jawbone, increased endosteal and periosteal perimeters and extensive trabeculation of femurs in *Ank* ^KI/KI^ mice shown by computed microtomography (μCT). This unexpected negative result is, however, consistent with normal serum/plasma levels of the intact/active form of FGF23 and PTH in *Ank* ^KI/KI^ mice and in CMD patients. In addition, FGF23 protein expression was unexpectedly normal in *Ank* ^KI/KI^ femoral cortical bone as shown by immunohistochemistry despite increased mRNA levels for *Fgf23*. Renal expression of genes involved in the FGF23 bone-kidney axis, including *mFgfr1, mKlotho, mNpt2a, mCyp24a1 and m1αOHase,* were comparable between *Ank* ^+/+^ and *Ank* ^KI/KI^ mice as shown by quantitative real-time PCR. Different from normal FGF23 and PTH, serum 25-hydroxyvitamin D was significantly lower in *Ank* ^KI/KI^ mice and vitamin D insufficiency was found in four out of seven CMD patients.

**Conclusions:**

Our data suggests that FGF23 signaling and Pi metabolism are not significantly affected in CMD and transiently low Pi level is not a major contributor to CMD.

## Background

Craniometaphyseal dysplasia (CMD), a rare genetic bone disorder, is characterized by progressive thickening of craniofacial bones and flared metaphyses of long bones [1]. CMD patients frequently suffer from hearing loss, visual impairment, facial paralysis and severe headaches due to consequences from neuronal compression as a result of hyperostosis of craniofacial bones [2, 3]. Treatment for CMD is currently limited to risky surgery to relieve neurologic symptoms, mainly because its pathogenesis is not well understood. A major hurdle for studying CMD and many other rare genetic bone disorders is the unavailability of specimens for research. We previously generated a knock-in (KI) mouse model by introducing a Phe377del mutation in *Ank* (progressive ankylosis gene), one of the most commonly identified mutations in CMD patients [4]. Homozygous *Ank* ^KI/KI^ mice replicate many features of CMD patients, including hyperostostic craniofacial bones, massive jaw bones, flared metaphyses with extensive trabeculation and fusion of middle ear bones [4]. *Ank* ^KI/KI^ mice differ from CMD patients in joint stiffness of elbows, knees, paws and vertebrae. This difference could be due to the autosomal dominant nature of ANK mutations in CMD patients and the mice we study are homozygous for the mutation. Heterozygous *Ank* ^+/KI^ mice do not have joint stiffness and only develop an intermediate skeletal CMD phenotype as they age [4]. Interestingly, *Ank* ^KI/KI^ mice present with significantly lower serum phosphate (Pi) levels at the age of 6 weeks but recover with age. *Ank* ^KI/KI^ mice display remarkably increased fibroblast growth factor 23 (*Fgf23*) mRNA expression in bones and develop hypomineralization of bones [5].

FGF23, a phosphaturic factor, is secreted by bone and acts in kidney to promote Pi wasting. It decreases 1,25-(OH)_2_D_3_ by reducing expression of *1-α hydroxylase*, *Npt2a/2c* (Na-Pi transporter) and by increasing the *24-hydroxylase* level upon binding to its receptor *Fgfr1* and co-receptor *Klotho* [6, 7]. Overexpression of FGF23 in mice leads to hypophosphatemia, hypomineralization, rickets and decreased 1,25-(OH)_2_D_3_, whereas ablation of FGF23 in mice leads to hyperphosphatemia, soft tissue calcification and increased 1,25-(OH)_2_D_3_ [8, 9]. Although much research has been done on FGF23, its role in the pathogenesis of CMD has never been addressed.

Mutations for the autosomal dominant form of CMD have been identified in the human progressive ankylosis gene (*ANKH,* encoding ANK), which is a known pyrophosphate (PPi) transporter [10, 11]. ANK controls Pi/PPi homeostasis together with plasma cell membrane glycoprotein (PC-1, encoded by ectonucleotide pyrophosphatase *Enpp1*), which generates PPi from nucleoside triphosphates (ATP) and tissue nonspecific alkaline phosphatase (TNAP, encoded by *Tnap*), which hydrolyzes extracellular PPi (ePPi) to generate Pi. The physiological concentration of ePPi in bone acts as a potent inhibitor of hydroxyapatite mineralization while supersaturation of ePPi leads to calcium pyrophosphate dehydrate (CPPD) crystal formation. Ank knock-out (*Ank* ^KO/KO^) mice exhibit joint stiffness due to the lack of PPi transporter, which results in excessive hydroxyapatite deposition in joints [12]. Pi is a major component of hydroxyapatite and promotes mineralization. A tightly controlled balance between extracellular Pi and PPi is required to maintain normal skeletal mineralization. CMD mutations in ANK result in reduced PPi-transport activity when overexpressed in oocytes [13]. How changes of Pi levels affect skeletal phenotypes in CMD and whether Pi can be used to treat CMD in mice or human have not been investigated.

The only source of Pi is from diet and Pi homeostasis is mainly regulated by three organs, parathyroid gland, kidney and bone, which produce parathyroid hormone (PTH), 1,25-(OH)_2_D_3_ (calcitriol, vitamin D) and FGF23, respectively. These three regulatory loops interact with one another. PTH stimulates Pi excretion and vitamin D production in kidney and stimulates FGF23 production in bone. PTH increases *Fgf23* expression in calvariae/femurs and serum FGF23 levels likely via activation of osteoblasts [14]. Low Pi and vitamin D in turn can inhibit PTH production. Vitamin D also increases Pi reabsorption and thus modulates bone formation and resorption. FGF23 increases Pi wasting and decreases vitamin D production. Vitamin D increases FGF23 production in bone and FGF23 acts as a counter-regulatory hormone to prevent the hyperphosphatemic effects of excess vitamin D [15, 16].

In order to study the involvement of Pi metabolism in CMD, we examined the levels of FGF23, PTH and 1,25-(OH)_2_D_3_ in a knock-in mouse model for CMD and in plasma collected from seven CMD patients. We fed mice with high Pi diet to evaluate whether the transiently lower serum Pi contributes to skeletal abnormalities in CMD and whether supplying increased dietary Pi ameliorates skeletal phenotypes of *Ank* ^KI/KI^ mice. Our results showed that Pi regulators were only mildly changed in CMD and high Pi diet did not correct hyperostosis in *Ank* ^KI/KI^ mice.

## Methods

### Mice

A mouse model with a deletion of TTC_1130–1132_ (phenylalanine 377) in exon 9 of *Ank* has been previously shown to replicate many features of human CMD [4]. Mice were bred from 129/Sv into a C57Bl/J6 background (N5) for skeletal analysis. High phosphate diet (1.5 %) (Harlan Teklad) was provided to mothers with litters after birth. Offspring were continuously fed with the modified diet for another 3 weeks after weaning. Thus, mice were fed with high Pi diet for a total of 6 weeks. Normal mouse chow contained 0.7 % phosphorus. The animal protocol (100782–1116) was approved by the Institutional Animal Care and Use Committee (IACUC) of University of Connecticut Health and all work was performed in an AAALAC accredited facility.

### Biochemical analysis

Blood of mice was obtained from the submandibular vein (Goldenrod animal lancet; Braintree Scientific) and collected in Microtainer tubes (Becton Dickinson). Fasting mouse sera were prepared from 3-, 10- and 16-week-old male *Ank* ^+/+^ and *Ank* ^KI/KI^ mice. Fasting human plasma was obtained from seven CMD patients. Research involving human subjects has been approved by the University of Connecticut Health Institutional Review Board (IRB number: 03-008-1) and written informed consent has been obtained from all participants. Concentrations of mouse intact FGF23 (mouse intact FGF23 ELISA kit; Kainos), mouse C-terminal FGF23 form (mouse FGF23 C-terminal ELISA kit; Immutopics), human intact FGF23 and C-terminal FGF23 form (human FGF23 intact ELISA kit, human FGF23 C-terminal ELISA kit; Immutopics), mouse/human 25-hydroxy vitamin D (25-hydroxy vitamin D kit; IDS), mouse and human intact PTH (mouse and human intact PTH ELISA kit; Immutopics) were determined according to manufacturers’ instructions.

### Immunohistochemistry

Femurs from 16-week-old male *Ank* ^+/+^ and *Ank* ^KI/KI^ mice were dissected, fixed in 4 % paraformaldehyde, decalcified in 14 % ethylenediaminetetraacetic acid, processed and embedded in paraffin. Series of 5 μm sections were collected using the central vein as marker. Briefly, the sections were pretreated with peroxidase blocking reagent, blocked with 1 % bovine serum albumin and 5 % normal goat serum for 30 min at room temperature and incubated with primary FGF23 antibody (1:50 dilution; MAB26291; R&D Systems). After washing, the sections were incubated with anti-rat HRP substrate and DAB (Vector Labs) and counterstained with methyl green. Isotype rat IgG2a control (R&D Systems) and femoral sections from FGF23 null mice (a kind gift of Dr. Beate Lanske) were used as negative controls for antibody specificity. The percentage of FGF23-positive cells over total osteocytes in cortical bones (from just below the growth plate to mid femurs) was determined.

### Quantitative real-time PCR (qPCR)

Total RNA from 16-week-old *Ank* ^+/+^ and *Ank* ^KI/KI^ kidney tissues was isolated with TRIzol (Invitrogen) according to manufacturer’s instructions. RNA was treated with DNase I (Invitrogen) and cDNA was synthesized using Superscript II reverse transcriptase (Invitrogen). qPCR using Power SYBR Master Mix (Life Technologies) was performed in an ABI-7300 thermocycler (Applied Biosystems). PCR efficiency was optimized and primer specificity was tested by melting curve analysis. Relative quantification of gene expression was determined using the ΔΔCt method by normalizing with 18S RNA. Data was presented as fold-increase relative to *Ank* ^+/+^ samples. PCR primer sequences are listed in Table 1.

**Table 1:** Amplification primers for qPCR

Gene	Forward primer	Reverse primer
*Fgfr1*	5'- CCAGTGCATCCATGAACTCTGGGGTTCTCC-3'	5'- GGTCACACGGTTGGGTTTGTCCTTATCCAG -3'
*Klotho*	5′- CTGGCTAAGGTTCAAGTACGGAGACCTCCC −3′	5'- GGAGCTGAGCGATCACTAAGTGAATACGCA -3'
*Npt2a*	5'- CTCATTGTGGGTGCCCAACATGATG -3'	5'- ACCATGTGTCTCCCACGGACTGGAAG -3'
*Cyp24a1*	5'- CTGCCCCATTGACAAAAGGC -3'	5'- CTCACCGTCGGTCATCAGC -3'
*1α*OHase	5'- TCAGATGTTTGCCTTTGCCC -3'	5'- TGGTTCCTCATCGCAGCTTC -3'
*18S*	5'-TTGACGGAAGGGCACCACCAG-3'	5'-GCACCACCACCCACGGAATCG-3'

### μCT analysis

Mandibles and femurs from mice fed with normal and high Pi diet for 6 weeks after birth were evaluated by μCT at the μCT Core Facility at UConn Health (mCT20; ScanCo Medical). For mandibles, the total volume (TV) and bone volume (BV) were measured. For femurs, the metaphyseal trabeculation and cortical bone parameters were measured as described previously [4].

### Statistical analysis

Statistical analysis was performed by Student’s t-test or analysis of variance (ANOVA) using Prism 5 software (GraphPad Software). Data was presented as mean ± standard deviation and statistically significant difference was considered for *p* < 0.05.

## Results

### Effects of high Pi diet on skeleton of *Ank*^KI/KI^ mice

We previously showed that serum Pi levels were decreased in 6-week-old *Ank* ^KI/KI^ mice fed with regular diet (*Ank* ^+/+^ = 6.622 ± 1.102 mg/dl, *n* = 10; *Ank* ^KI/KI^ = 5.494 ± 0.893 mg/dl, *n* = 11; *p* < 0.05) and were normal in 10-week-old *Ank* ^KI/KI^ mice [4]. To examine whether dietary Pi can rescue the CMD-like skeletal phenotype, we fed high Pi diet (1.5 %) to *Ank* ^+/+^ and *Ank* ^KI/KI^ mice continuously for 6 weeks after birth. Serum Pi levels in mice fed with high Pi diet for 6 weeks did increase (*Ank* ^+/+^ = 7.554 ± 0.745 mg/dl, *n* = 9; *Ank* ^KI/KI^ = 6.799 ± 0.771 mg/dl, *n* = 7) but no significant difference between *Ank* ^+/+^ and *Ank* ^KI/KI^ mice was detected.

**Fig. 1:**
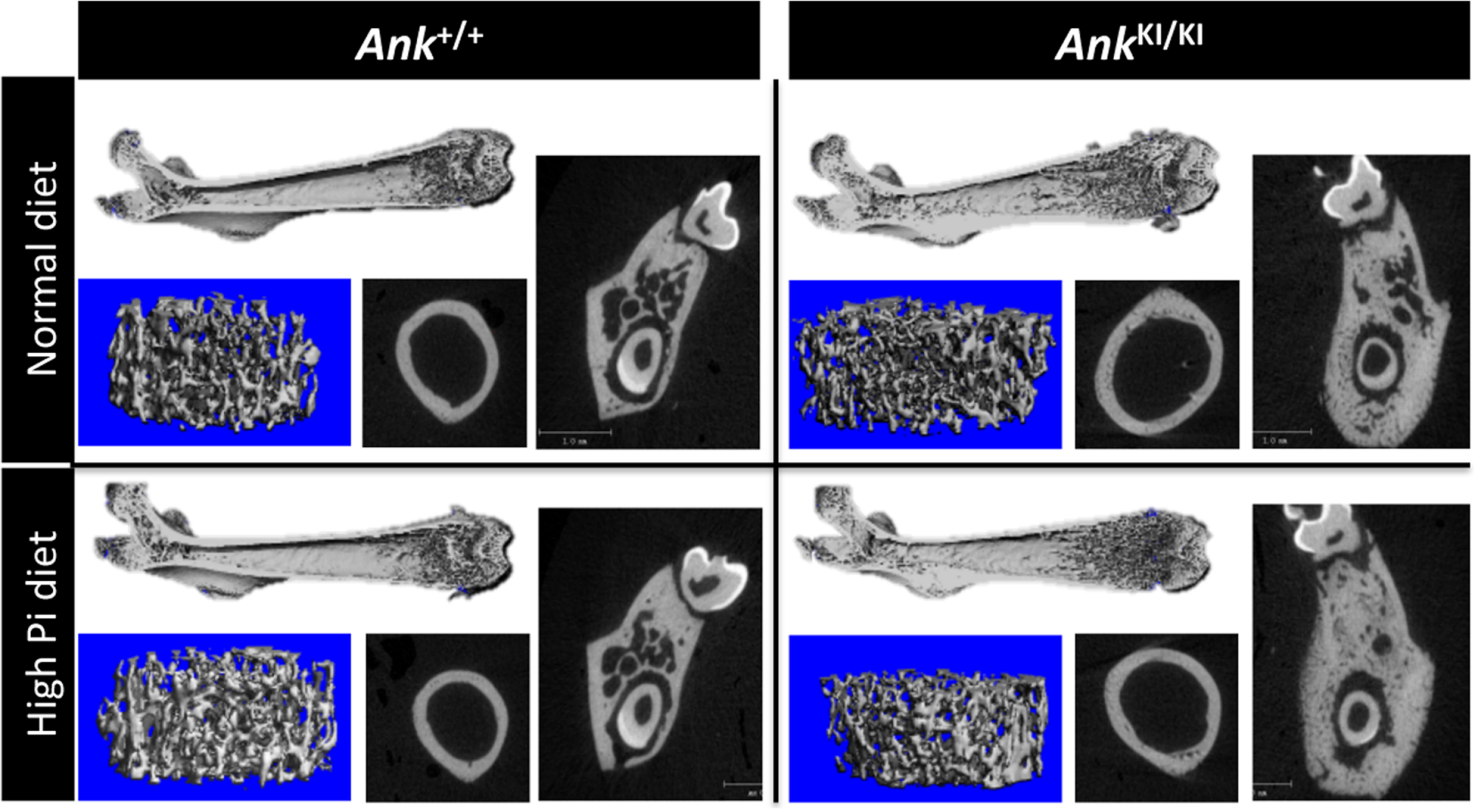
μCT images of femurs and mandibles. Representative μCT 3D reconstruction images of femurs and sagittal planes through furcation of first molar of mandibles from 6-week-old male *Ank* ^+/+^ and *Ank* ^KI/KI^ mice fed with normal or high Pi diet. (*Ank* ^+/+^ mice with normal diet *n* = 4; *Ank* ^KI/KI^ mice with normal diet *n* = 5; *Ank* ^+/+^ mice with high Pi diet, *n* = 7; *Ank* ^KI/KI^ mice with high Pi diet, *n* = 8)

High Pi diet in general resulted in a tendency of more trabecular bone, decreased sub-periosteal and sub-endosteal areas, decreased cortical porosity in femurs and increased bone volume in mandibles of *Ank* ^+/+^ and *Ank* ^KI/KI^ mice. Differences between *Ank* ^+/+^ and *Ank* ^KI/KI^ femurs in metaphyseal trabeculation, measured by bone volume fraction (BVF), trabecular number, trabecular thickness and trabecular space, were abolished by high Pi diet mainly because of increased bone volume in *Ank* ^+/+^ mice. The extended trabeculation extending into the diaphysis of *Ank* ^KI/KI^ club-shaped femurs remained the same with the high Pi diet (Fig. 1). These mice continued to have massive jawbones, club-shaped femurs, extensive trabeculation, increased cortical porosity and decreased tissue density of femurs when compared to *Ank* ^+/+^ mice. Skeletal features analyzed by μCT are summarized in Table 2 and Fig. 1.

**Table 2:** μCT analysis of 6-week-old male *Ank* ^+/+^ and *Ank* ^KI/KI^ mice fed with normal and high Pi diet

	Normal Pi		High Pi	
Parameters	*Ank* ^+/+^(*n* = 4)	*Ank* ^KI/KI^ (*n* = 5)	*Ank* ^+/+^(*n* = 7)	*Ank* ^KI/KI^ (*n* = 8)
Mandible				
Total volume (mm^3^)	0.28 ± 0.01	0.42 ± 0.02^c^	0.31 ± 0.01^a^	0.51 ± 0.04^b, c^
Bone volume (mm^3^)	0.18 ± 0.02	0.31 ± 0.03^c^	0.23 ± 0.001^a^	0.44 ± 0.04^b, c^
BVF (%)	66.5 ± 0.2	77.4 ± 2.2^c^	74 ± 2.3^a^	86 ± 0.01^b, c^
Femur trabecular bone (metaphyses)				
BVF (%)	5.9 ± 0.4	9.1 ± 0.4^c^	9.7 ± 3.1	9.9 ± 2.3
Trabecular number (N/mm)	4 ± 0.01	5.3 ± 0.12^c^	5.27 ± 0.81^a^	5.66 ± 0.48
Trabecular thickness (μm)	34 ± 2.4	37.6 ± 0.86^c^	36.2 ± 2.5	36.6 ± 2.2
Trabecular Spacing (μm)	248.53 ± 19.49	186.9 ± 5.62^c^	193.19 ± 32.10^a^	173.35 ± 15.68
Femur cortical bone (diaphyses)				
Subperiosteal area (mm^2^)	1.64 ± 0.11	2.26 ± 0.22^c^	1.20 ± 0.32^a^	1.7 ± 0.10^b,^ ^c^
Subendosteal area (mm^2^)	1.05 ± 0.06	1.46 ± 0.16^c^	0.88 ± 0.14	1.18 ± 0.09^c^
Cortical porosity (%)	0.4 ± 0.2	1.95 ± 0.01^c^	0.4 ± 0.23	1.16 ± 0.01^c^
Tissue density (mg/cm^3^ HA)	1135.0 ± 30.9	1125.0 ± 13.5^c^	1141.6 ± 11.1	1106.4 ± 16.1^c^

BVF: bone volume fraction (bone volume/total volume)(%); ^a^ *p* < 0.05: statistical significance between *Ank* ^+/+^ mice fed with normal and Pi diet;^b^ *p* < 0.05: statistical significance between *Ank* ^KI/KI^ mice fed with normal and Pi diet;^c^ *p* < 0.05: statistical significance between *Ank* ^+/+^ and *Ank* ^KI/KI^ mice fed with the same diet. Data are mean ± SD

### FGF23 expression in *Ank*^KI/KI^ mice

**Fig. 2:**
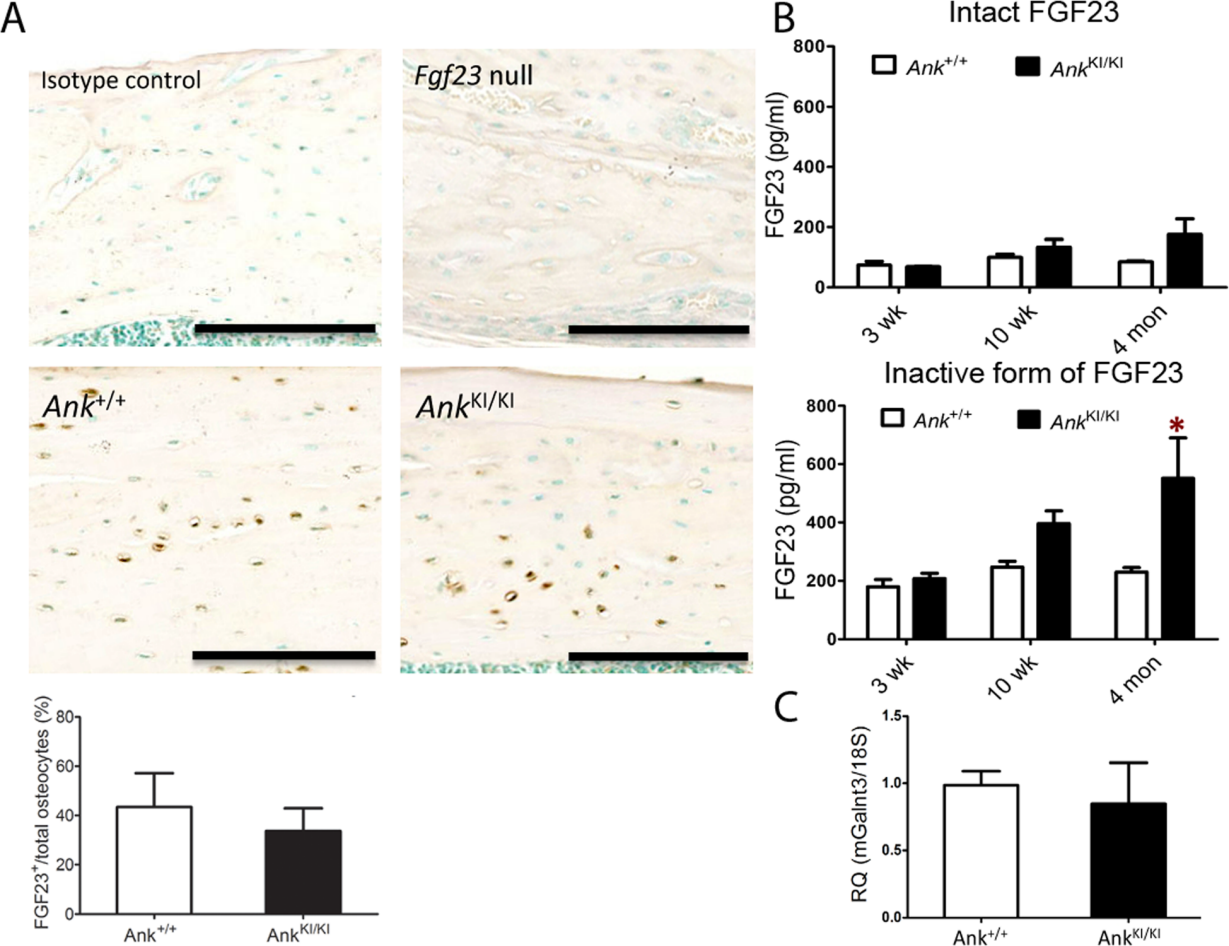
FGF23 in *Ank* ^KI/KI^ mice. **a** Immunohistochemistry showed no significant difference of FGF23 in femoral cortical bones from 16-week-old *Ank* ^+/+^ and *Ank* ^KI/KI^ mice. Femurs stained with isotype IgG antibody and femurs from *Fgf23* null mice were used as negative controls. FGF23-positive cells stained *brown*. Nuclei are counterstained with methyl *green*. Histogram shows numbers of FGF23-positive cells normalized to total number of osteocytes. *Ank* ^+/+^ *n* = 5, *Ank* ^KI/KI^ *n* = 7; Scale bar = 100 μm. **b** ELISA assays of intact form of FGF23 in serum (top panel) and C-terminal form of FGF23 (bottom panel) in 3-, 10- and 16-week-old *Ank* ^+/+^ and *Ank* ^KI/KI^ mice. 3-week-old mice: *Ank* ^+/+^ *n* = 7, *Ank* ^KI/KI^ *n* = 5; 10-week-old *Ank* ^+/+^ *n* = 10, *Ank* ^KI/KI^ =9; 16-week-old *Ank* ^+/+^ *n* = 5, *Ank* ^KI/KI^ *n* = 5. **c** qPCR of *Galnt3* expression in femoral bones of 16-week-old *Ank* ^+/+^ and *Ank* ^KI/KI^ mice. Data are mean ± S.D. * *p* < 0.05

To determine whether increased *Fgf23* mRNA led to increased FGF23 protein levels, we performed immunohistochemistry with FGF23 antibodies on femoral sections from 16-week-old mice. To confirm specificity of the FGF23 antibody, we included femurs from FGF23 null mice as negative controls. The percentage of FGF23-positive cells normalized to total osteocytes in cortical bones was determined. Unexpectedly, there was no significant difference in the percentage of FGF23-positive osteocytes in femoral cortical bones between *Ank* ^+/+^ and *Ank* ^KI/KI^ mice (Fig. 2a).

Intact FGF23 protein will become inactive after being cleaved. To examine whether the discrepancy between mRNA and protein levels of FGF23 in *Ank* ^KI/KI^ mice is due to increased proteolytic processing of FGF23, we investigated FGF23 serum levels in male mice at ages 3, 10 and 16 weeks by ELISA using antibodies recognizing the full-length FGF23, which is the biologically active form, or antibodies recognizing both full-length and C-terminal fragments of FGF23. Cleaved FGF23 is the inactive form. *Ank* ^KI/KI^ mice showed a trend but no statistical significance in the increase of intact FGF23 but a significant increase in the inactive C-terminal fragment of FGF23 suggesting that more FGF23 is processed in *Ank* ^KI/KI^ mice. This significant increase is only observed in 16-week-old *Ank* ^KI/KI^ mice but not at ages of 3 or 10 weeks (Fig. 2b).

Galnt3, a GalNac-transferase, prevents the proteolytic processing of FGF23 by mediating the glycosylation of a furin-like convertase recognition sequence in FGF23 [17]. To examine whether increased serum C-terminal FGF23 in *Ank* ^KI/KI^ mice is due to changes in *Galnt3* gene expression, we examined *Galnt3* mRNA expression by qPCR and found comparable levels of *Galnt3* between *Ank* ^+/+^ and *Ank* ^KI/KI^ mice (Fig. 2c). This finding indicates that the increased C-terminal form of FGF23 in *Ank* ^KI/KI^ mice is likely *Galnt3*-independent. The activity of GALNT3 protein or other mechanisms regulating post-translational modification of FGF23 will need further investigation.

### Renal expression of genes involved in FGF23 bone-kidney axis

**Fig. 3:**
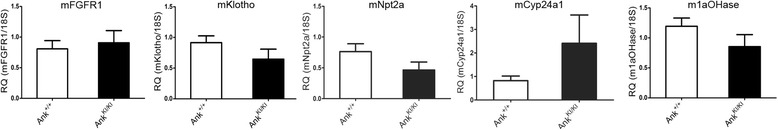
Comparable expression of genes involved in FGF23 bone-kidney axis. qPCR of *mFgfr1, mKlotho, mNpt2a, mCyp24a1 and m1αOHase* expression in kidney from 16-week-old *Ank* ^+/+^ and *Ank* ^KI/KI^ mice. Data are mean ± SD. No significant differences were detected. RQ: relative quantification

The kidney plays an important role in regulating Pi homeostasis and is a target of FGF23. To determine if the increasing trend of the active form of FGF23 in *Ank* ^KI/KI^ serum, though not significant, can lead to changes within kidneys, we first examined the ratio of kidney weight over total body weight in *Ank* ^+/+^ and *Ank* ^KI/KI^ mice. *Ank* ^KI/KI^ mice showed increased kidney/body weight ratio compared to *Ank* ^+/+^ mice at the age of 16 weeks, however, this was likely due to remarkably decreased body weight rather than kidney hypertrophy since no abnormal morphologic structures in kidneys of *Ank* ^KI/KI^ mice were observed by H&E staining (Table 3; data not shown). To study whether the renal expression of genes targeted by FGF23 are affected in *Ank* ^KI/KI^ mice, qPCR with specific primers to *Fgfr1* (FGF23 receptor)*, Klotho* (FGF23 co-receptor)*, Npt2a* (sodium phosphate co-transporter)*, Cyp24a1* (1α-hydroxy-vitamin D 24-hydroxylase) and *Cyp27b1* (*1a(OH)ase*, 1α-hydroxylase) was performed. Kidney mRNA isolated from both 10-week-old (data not shown) and 16-week-old *Ank* ^+/+^ and *Ank* ^KI/KI^ mice was examined. Our results showed a trend of decreased expression of *1a(OH)ase, Npt2a*, *Klotho* and a trend of increased expression of *Cyp24a1* in 16-week-old *Ank* ^*KI/KI*^ mice, however, none of them was statistically significant (Fig. 3).

**Table 3:** Body weight, kidney weight (sum of right and left kidneys) and the ratio of kidney to body weight in *Ank* ^+/+^ and *Ank* ^KI/KI^ mice

	*Ank* ^+/+^	*Ank* ^KI/KI^
BW 6 weeks	22.83 ± 1.91 (*n* = 7)	20.83 ± 2.27 (*n* = 8)
BW 10 weeks	27.35 ± 2.94 (*n* = 12)	23.46 ± 1.57^a^ (*n* = 10)
BW 20 weeks	38.37.2 ± 6.3 (*n* = 10)	20.54 ± 2.96^b^ (*n* = 6)
KW 6 weeks	359.5 ± 26.8	334.1 ± 53.2
KW 10 weeks	336.3 ± 64.0	297.9 ± 72.7
KW 20 weeks	455.0 ± 68.9	329.5 ± 66.5^b^
KW/BW 6 weeks	15.75 ± 0.15	16.08 ± 2.17
KW/BW 10 weeks	12.45 ± 2.60	12.69 ± 3.06
KW/BW 20 weeks	12.01 ± 1.54	15.99 ± 2.13^b^

BW: body weight (g); KW: kidney weight (mg); ^a^ *p* < 0.05, ^b^ *p* < 0.001 between *Ank* ^+/+^ and *Ank* ^KI/KI^ mice

### PTH and 25(OH) vitamin D in mouse serum

Parathyroid hormone (PTH), 1,25-(OH)_2_D_3_ (calcitriol) and FGF23 interact to maintain Pi homeostasis. We previously reported lower Pi serum levels and normal PTH in *Ank* ^KI/KI^ mice at the age of 6 weeks. However, the lower Pi serum level was within normal range as mice aged to 10 weeks and older [5]. Because we found an increasing tendency of the active form of FGF23 in *Ank* ^KI/KI^ mice as they aged, we decided to measure serum PTH and 25-hydroxy vitamin D at ages of 10 weeks and 16 weeks. 25-hydroxy vitamin D (25OHD) is a pre-hormone that is converted into 1,25-(OH)_2_D_3_ in the kidneys and is considered the best indicator of vitamin D status. Serum PTH levels were comparable between *Ank* ^+/+^ and *Ank* ^KI/KI^ mice (10 weeks PTH, *Ank* ^+/+^:*Ank* ^KI/KI^ = 238.25 ± 135.66 (pg/ml) (*n* = 19) : 217.83 ± 99.01(pg/ml) (*n* = 16); 16 weeks PTH, *Ank* ^+/+^:*Ank* ^KI/KI^ = 222.13 ± 66.8 (pg/ml) (*n* = 5) : 242.98 ± 59.8 (pg/ml) (*n* = 5)). 25OHD was significantly reduced at the age of 10 weeks (*Ank* ^+/+^:*Ank* ^KI/KI^ = 41.5 ± 6.61 (ng/ml) (*n* = 13) : 32.01 ± 2.96 (ng/ml) (*n* = 12), *p* < 0.05) but not at 16 weeks (*Ank* ^+/+^: *Ank* ^KI/KI^ = 41.21 ± 7.42 (ng/ml) (*n* = 9) : 36.83 ± 5.79 (ng/ml) (*n* = 12), *p* = 0.16).

### Biochemical analysis of plasma from CMD patients

We determined plasma levels of hormones that are affecting or are affected by Pi homeostasis in seven CMD patients (Table 4). PTH and the intact and C-terminal forms of FGF23 were normal in CMD patients. Four out of seven CMD patients had insufficiency or deficiency of vitamin D measured by 25OHD.

**Table 4:** Biochemical analysis of plasma from CMD patients

Case	Ank mutation	Age	Gender	PTH (pg/ml)	25OHD (ng/ml)	i-FGF23 (pg/ml)	c-FGF23 (RU/ml)
1	F377del	45	F	63.14	32.90	13.42	61.85
2	F377del	17	F	37.38	40.92	19.84	59.02
3	F377del	14	F	31.41	34.26	14.64	50.83
4	F377del	9	F	28.78	**29.68**	13.71	14.25
5	S375del	10	F	35.57	**13.4**	33.16	31.95
6	S375del	48	F	26.75	**18.56**	13.51	30.66
7	C331 → R	18	M	16.53	**23.82**	10.02	63.25

i-FGF23: intact FGF23; c-FGF23: C-terminal form of FGF23; Normal range of human hormones: 1) human PTH: 12–65 pg/ml; 2) human i-FGF23: 10–50 pg/ml; 3) human c-FGF23: <120-150 RU/ml. 4) human vitamin D insufficiency: 21–29 ng/ml; vitamin D deficiency < 20 ng/ml. Bold number indicated cases with vitamin D insufficiency or deficiency

## Discussion

Pi metabolism plays an important role in the regulation of skeletogenesis [18]. Serum Pi levels in CMD patients have been reported as transiently lower or normal [19–21]. Consistently, we observed lower serum Pi levels in *Ank* ^KI/KI^ mice at the age of 6 weeks but serum Pi levels were normal in 10-week-old *Ank* ^KI/KI^ mice [5]. Interestingly, mild hypophosphatemia has also been associated with patients expressing a homozygous *ANKH* missense mutation (Leu244Ser) and presenting mental retardation, hearing loss, ankylosis, periarticular ligament ossification, enthesopathy, and dentinogenesis imperfecta [22].

In order to study whether Pi metabolism is abnormal in mice and patients with CMD and whether dietary Pi affects the bone phenotype in the CMD mouse model we utilized high Pi diet in mice to examine the effects of Pi on the bone phenotype. We compared *Ank* ^+/+^ and *Ank* ^KI/KI^ littermates fed with normal mouse chow and high Pi diet. We detected increased serum Pi levels after feeding mice with high Pi diet for 6 weeks. As expected, high Pi diet increases the bone volume in general because Pi is required for hydroxyapatite (calcium phosphate) formation. Pi can also affect osteoclast activity although its effects remain controversial. It has been shown that high Pi decreased osteoclastogenesis *in vitro* [23]. On the other hand, high Pi diet has been shown to increase osteoclast numbers in the *Hyp* mice [24]. Our μCT data showed that phenotypic differences between *Ank* ^+/+^ and *Ank* ^KI/KI^ mice, including the club-shaped femurs, increased cortical porosity and hyperostosis of mandibles in *Ank* ^KI/KI^ mice, did not change with high Pi diet. We therefore, did not further perform static or dynamic histomorphometry with these bones. This data indicates that dietary Pi supplement is not efficient in correcting the CMD phenotype and may be counterproductive.

We examined FGF23 and the FGF23-mediated bone-kidney axis because we previously found significantly increased *Fgf23* mRNA levels in calvarial and femoral bones of *Ank* ^KI/KI^ mice [5]. FGF23 has been associated with a number of human diseases, including X-linked hypophosphatemia (XLH), autosomal dominant hypophosphatemic rickets (ADHR), chronic kidney disease (CKD), familial tumoral calcinosis, McCune-Albright syndrome, and fibrous dysplasia of bone [25–29]. Unexpectedly, we did not detect significant differences in FGF23 protein expression in femoral bones or of the active form of FGF23 in serum from *Ank* ^+/+^ and *Ank* ^KI/KI^ mice. We also did not observe significant pathological changes in renal morphology or in abnormal mRNA expression of FGF23-target genes in *Ank* ^KI/KI^ kidneys. Although the mechanisms that would explain the discrepancy between *Fgf23* mRNA and FGF23 protein levels are yet unknown, our data suggests that increased *Fgf23* transcripts are unlikely the primary factor contributing to the skeletal phenotype in CMD.

Normalization of serum Pi levels in *Ank* ^KI/KI^ mice as they age suggests that a feedback mechanism may be involved to correct the decreased Pi levels as *Ank* ^KI/KI^ mice age [5]. We therefore examined levels of FGF23, PTH and 1,25-(OH)_2_D_3_ which are regulators known to control Pi homeostasis [30, 31]. The active form 1,25-(OH)_2_D_3_ results from formation of cholecalciferol in the skin by photolysis of 7-dehydrocholesterol under sunlight exposure followed by hydroxylation of cholecalciferol to 25-hydroxy vitamin D (25OHD) in the liver. 25OHD is further hydroxylated in the kidney to form the active form 1,25-(OH)_2_D_3_. Although commercial kits are available to measure 1,25-(OH)_2_D_3_, the vitamin D status is best determined by 25OHD levels [32, 33]. This is because 25OHD has a half-life of approximately 3 weeks whereas 1,25(OH)_2_D_3_ has a half-life of only 4–6 h. 25OHD is unregulated and can reflect substrate availability. On the other hand, 1,25-(OH)_2_D_3_ is highly regulated and therefore provides limited information about the vitamin D status. In both, CMD mice and patients with CMD, we found normal intact FGF23 and PTH levels. 25OHD was transiently lower in *Ank* ^KI/KI^ mice at the age of 10 weeks but became comparable to *Ank* ^+/+^ mice as they aged to 16 weeks. Four out of seven CMD patients showed vitamin D insufficiency or deficiency. Lower vitamin D levels may be associated with insufficient nutritional uptake, reduced sun exposure or the status of vitamin D binding protein (DBP). A recent study suggests that a specific type of nucleotide polymorphisms in *Gc*, the gene encoding DBP, is associated with the highest 25OHD concentration compared to other lower affinity *Gc* variants in West African children [34]. Further studies will be needed to investigate mechanisms leading to decreased vitamin D levels in CMD, such as whether DBP status is affected by CMD ANKH mutations. Due to the limited number of CMD cases in this study no conclusion can be drawn based on biochemical analyses.

There are several limitations to this study. One limitation is that we have samples from only seven CMD patients of different gender and age because CMD is a very rare disorder. Secondly, it has been challenging to recruit healthy age and sex matched subjects as controls for the individual assays. We thus compared our data to well-accepted normal ranges of FGF23, PTH and 25OHD. Lastly, the mechanisms of increased cleavage of FGF23 and transiently decreased vitamin D in *Ank* ^KI/KI^ mice are still unknown. Nonetheless, this project is significant as we show that Pi metabolism is not significantly affected and thus is unlikely a major contributor to CMD. A tight balance between Pi and PPi is important for regulation of skeletal modeling and remodeling. CMD mutant ANK has been reported to reduce PPi transporting activity [13]. Although we found that Pi metabolism or Pi levels are not severely altered in CMD mice or patients, adjusting the Pi/PPi ratio might ameliorate skeletal abnormalities of CMD. Clinically, patients can reduce serum Pi levels by avoiding foods that are high in Pi or by Pi binders, such as aluminum salts, calcium carbonate, calcium acetate and lanthanum carbonate. Future studies will focus on whether a low Pi diet in *Ank* ^KI/KI^ mice is beneficial for correcting a possibly altered Pi/PPi ratio in CMD, which may be caused by decreased PPi.

## Conclusions

Our data indicate that the bone-kidney axis of FGF23 is not a primary contributor to the pathology of CMD. Levels of circulating PTH and active form of FGF23 are within the normal range in *Ank* ^KI/KI^ mice and CMD patients. High Pi diet does not appear to correct the bone phenotype of CMD.

## Abbreviations

ANK/ANKH: progressive ankylosis gene
CMD: craniometaphyseal dysplasia
FGF23: fibroblast growth factor 23
Pi: phosphate
PPi: pyrophosphate
PTH: parathyroid hormone

## References

[1] Jackson WP (1954). Metaphyseal dysplasia, epiphyseal dysplasia, diaphyseal dysplasia, and related conditions. I. Familial metaphyseal dysplasia and craniometaphyseal dysplasia; their relation to leontiasis ossea and osteopetrosis; disorders of bone remodeling. AMA Arch Intern Med.

[2] Cheung VG, Boechat MI, Barrett CT (1997). Bilateral choanal narrowing as a presentation of craniometaphyseal dysplasia. J Perinatol.

[3] Richards A (1996). Craniometaphyseal and craniodiaphyseal dysplasia, head and neck manifestations and management. J Laryngol Otol.

[4] Chen IP (2009). Introduction of a Phe377del mutation in ANK creates a mouse model for craniometaphyseal dysplasia. J Bone Miner Res.

[5] Chen IP (2011). A Phe377del mutation in ANK leads to impaired osteoblastogenesis and osteoclastogenesis in a mouse model for craniometaphyseal dysplasia (CMD). Hum Mol Genet.

[6] Shimada T (2004). FGF-23 is a potent regulator of vitamin D metabolism and phosphate homeostasis. J Bone Miner Res.

[7] Urakawa I (2006). Klotho converts canonical FGF receptor into a specific receptor for FGF23. Nature.

[8] Bai X (2004). Transgenic mice overexpressing human fibroblast growth factor 23 (R176Q) delineate a putative role for parathyroid hormone in renal phosphate wasting disorders. Endocrinology.

[9] Shimada T (2004). Targeted ablation of Fgf23 demonstrates an essential physiological role of FGF23 in phosphate and vitamin D metabolism. J Clin Invest.

[10] Reichenberger E (2001). Autosomal dominant craniometaphyseal dysplasia is caused by mutations in the transmembrane protein ANK. Am J Hum Genet.

[11] Nurnberg P (2001). Heterozygous mutations in ANKH, the human ortholog of the mouse progressive ankylosis gene, result in craniometaphyseal dysplasia. Nat Genet.

[12] Gurley KA (2006). Mineral formation in joints caused by complete or joint-specific loss of ANK function. J Bone Miner Res.

[13] Gurley KA, Reimer RJ, Kingsley DM (2006). Biochemical and genetic analysis of ANK in arthritis and bone disease. Am J Hum Genet.

[14] Kawata T (2007). Parathyroid hormone regulates fibroblast growth factor-23 in a mouse model of primary hyperparathyroidism. J Am Soc Nephrol.

[15] Liu S (2006). Fibroblast growth factor 23 is a counter-regulatory phosphaturic hormone for vitamin D. J Am Soc Nephrol.

[16] Kolek OI (2005). 1alpha,25-Dihydroxyvitamin D3 upregulates FGF23 gene expression in bone: the final link in a renal-gastrointestinal-skeletal axis that controls phosphate transport. Am J Physiol Gastrointest Liver Physiol.

[17] Frishberg Y (2007). Hyperostosis-hyperphosphatemia syndrome: a congenital disorder of O-glycosylation associated with augmented processing of fibroblast growth factor 23. J Bone Miner Res.

[18] Zhang R (2011). Unique roles of phosphorus in endochondral bone formation and osteocyte maturation. J Bone Miner Res.

[19] Dutra EH (2012). Two novel large ANKH deletion mutations in sporadic cases with craniometaphyseal dysplasia. Clinical Genetics.

[20] Mintz S, Velez I (2004). Craniometaphyseal dysplasia associated with obstructive sleep apnoea syndrome. Dentomaxillofacial Radiol.

[21] Yamamoto T (1993). Bone marrow-derived osteoclast-like cells from a patient with craniometaphyseal dysplasia lack expression of osteoclast-reactive vacuolar proton pump. J Clin Investig.

[22] Morava E (2011). Autosomal recessive mental retardation, deafness, ankylosis, and mild hypophosphatemia associated with a novel ANKH mutation in a consanguineous family. J Clin Endocrinol Metab.

[23] Kanatani M (2003). Effect of high phosphate concentration on osteoclast differentiation as well as bone-resorbing activity. J Cell Physiol.

[24] Hayashibara T (2007). Regulation of Osteoclast Differentiation and Function by Phosphate: Potential Role of Osteoclasts in the Skeletal Abnormalities in Hypophosphatemic Conditions. J Bone Miner Res.

[25] A gene (PEX) with homologies to endopeptidases is mutated in patients with X-linked hypophosphatemic rickets. The HYP Consortium. Nat Genet. 1995;11(2):130–6. 10.1038/ng1095-130 7550339

[26] Consortium A (2000). Autosomal dominant hypophosphataemic rickets is associated with mutations in FGF23. Nat Genet.

[27] Larsson T (2003). Circulating concentration of FGF-23 increases as renal function declines in patients with chronic kidney disease, but does not change in response to variation in phosphate intake in healthy volunteers. Kidney Int.

[28] Larsson T (2005). A novel recessive mutation in fibroblast growth factor-23 causes familial tumoral calcinosis. J Clin Endocrinol Metab.

[29] Riminucci M (2003). FGF-23 in fibrous dysplasia of bone and its relationship to renal phosphate wasting. J Clin Invest.

[30] Potts JT (2005). Parathyroid hormone: past and present. J Endocrinol.

[31] Torres PA, De Brauwere DP (2011). Three feedback loops precisely regulating serum phosphate concentration. Kidney Int.

[32] Holick MF (1990). The use and interpretation of assays for vitamin D and its metabolites. J Nutr.

[33] Iqbal SJ (1994). Vitamin D metabolism and the clinical aspects of measuring metabolites. Ann Clin Biochem.

[34] Braithwaite VS (2015). Vitamin D binding protein genotype is associated with plasma 25OHD concentration in West African children. Bone.

